# Evolutionary engineering and molecular characterization of cobalt-resistant *Rhodobacter sphaeroides*

**DOI:** 10.3389/fmicb.2024.1412294

**Published:** 2024-06-27

**Authors:** Güneş Atay, Can Holyavkin, Hanay Can, Mevlüt Arslan, Alican Topaloğlu, Massimo Trotta, Zeynep Petek Çakar

**Affiliations:** ^1^Department of Molecular Biology and Genetics, Faculty of Science and Letters, Istanbul Technical University, İstanbul, Türkiye; ^2^Dr. Orhan Öcalgiray Molecular Biology, Biotechnology and Genetics Research Center (İTÜ-MOBGAM), Istanbul Technical University, İstanbul, Türkiye; ^3^IPCF-CNR Istituto per I processi Chimico-Fisici, Consiglio Nazionale delle Ricerche, Bari, Italy

**Keywords:** adaptive laboratory evolution, cobalt resistance, evolutionary engineering, genomic analysis, *Rhodobacter sphaeroides*, stress resistance

## Abstract

With its versatile metabolism including aerobic and anaerobic respiration, photosynthesis, photo-fermentation and nitrogen fixation, *Rhodobacter sphaeroides* can adapt to diverse environmental and nutritional conditions, including the presence of various stressors such as heavy metals. Thus, it is an important microorganism to study the molecular mechanisms of bacterial stress response and resistance, and to be used as a microbial cell factory for biotechnological applications or bioremediation. In this study, a highly cobalt-resistant and genetically stable *R. sphaeroides* strain was obtained by evolutionary engineering, also known as adaptive laboratory evolution (ALE), a powerful strategy to improve and characterize genetically complex, desired microbial phenotypes, such as stress resistance. For this purpose, successive batch selection was performed in the presence of gradually increased cobalt stress levels between 0.1–15 mM CoCl_2_ for 64 passages and without any mutagenesis of the initial population prior to selection. The mutant individuals were randomly chosen from the last population and analyzed in detail. Among these, a highly cobalt-resistant and genetically stable evolved strain called G7 showed significant cross-resistance against various stressors such as iron, magnesium, nickel, aluminum, and NaCl. Growth profiles and flame atomic absorption spectrometry analysis results revealed that in the presence of 4 mM CoCl_2_ that significantly inhibited growth of the reference strain, the growth of the evolved strain was unaffected, and higher levels of cobalt ions were associated with G7 cells than the reference strain. This may imply that cobalt ions accumulated in or on G7 cells, indicating the potential of G7 for cobalt bioremediation. Whole genome sequencing of the evolved strain identified 23 single nucleotide polymorphisms in various genes that are associated with transcriptional regulators, NifB family-FeMo cofactor biosynthesis, putative virulence factors, TRAP-T family transporter, sodium/proton antiporter, and also in genes with unknown functions, which may have a potential role in the cobalt resistance of *R. sphaeroides*.

## 1 Introduction

Photosynthetic purple non-sulfur bacteria are commonly used as model organisms to study bacterial photosynthesis (Allen et al., 1988; Yeates et al., 1988; Grattieri et al., 2022). They are also industrially important microorganisms because of their ability to produce hydrogen (H_2_) (Gökçe et al., 2012, 2017; Vasiliadou et al., 2018; Li et al., 2024), and their potential applications in bioremediation and bio-sensing (Buscemi et al., 2022; Grattieri et al., 2022). Among photosynthetic purple non-sulfur bacteria, *Rhodobacter sphaeroides* is particularly important, as it has a high adaptation ability that allows it to grow in diverse nutritional and environmental conditions, including heavy-metal-polluted environments (Volpicella et al., 2014). The versatile metabolism of *R. sphaeroides* includes growth by aerobic and anaerobic respiration, photosynthesis under anaerobic conditions in the presence of light and photo-fermentation (Kiley and Kaplan, 1988; Martínez-Luque et al., 1991; Gupta et al., 2024). Upon decreasing oxygen levels, it can easily switch to photosynthetic growth by remodeling the intracellular membrane and producing pigments required for capturing light energy (Imam et al., 2014). *R. sphaeroides* is able to fix CO_2_ and N_2_, and produce H_2_ and polyhydroxybutyrate (PHB) (Mackenzie et al., 2007; Imam et al., 2013). It has a significant biotechnological potential regarding bio-based production of chemicals and fuels (Zeilstra-Ryalls et al., 1998; Mackenzie et al., 2007; Burger et al., 2017; Henry et al., 2020).

Various studies have shown that *R. sphaeroides* can tolerate metal stress, sequester metal ions and reduce oxyanions (Moore and Kaplan, 1992; Bebien et al., 2001; Giotta et al., 2006), as reported previously (Volpicella et al., 2014). Giotta et al. (2006) have shown that *R. sphaeroides* is highly tolerant to Hg^2+^, Cu^2+^, Fe^2+^, Ni^2+^, Co^2+^, MoO_4_^2−^, and CrO_4_^2−^, particularly to Co^2+^, MoO_4_^2−^ and Fe^2+^. The EC_50_ value for Co^2+^ was 0.8 mM, and a consistent biomass yield was obtained even at high cobalt concentrations (5 mM), indicating high cobalt tolerance for *R. sphaeroides* (Giotta et al., 2006). These findings have led to more detailed investigations of the response and resistance of *R. sphaeroides* to cobalt stress.

Cobalt is an industrially important element with magnetic properties and a variety of applications, including refining of alloys, production of gas turbines, jet engines, electrochemical materials and permanent magnets. It is also used in catalysts, paints, varnishes, pigments, inks, ceramics and surgical implants (Stadler and Schweyen, 2002). Most importantly, cobalt has been increasingly used in the cathodes of lithium-ions batteries of highly demanded electric vehicles, to tackle the global warming issue (Lee and Manthiram, 2022). In line with the transition of energy system to clean energy, the global cobalt industry chain has been in focus for risk management and sustainability purposes (Shi et al., 2022).

In addition to its industrial importance, cobalt also has important biological functions. As an essential micronutrient, it is used as a cofactor of vitamin B12 (cobalamin), and in the catalysis of important enzymes in microorganisms, plants and animals (Battersby, 1994; Kobayashi and Shimizu, 1999). However, as a transition metal, cobalt is toxic to living organisms at high concentrations (Çakar et al., 2009; Pisani et al., 2009; Belviso et al., 2013). In a yeast (*Saccharomyces cerevisiae*) reference strain CEN.PK 113-7D; 2.5 mM CoCl_2_ resulted in 50% inhibition of growth, and no growth was observed at CoCl_2_ concentrations higher than 5 mM (Çakar et al., 2009). The bacterium *Pseudomonas aeruginosa* could tolerate Co^2+^ concentrations lower than 0.8 mM (Hassen et al., 1998). In *R. sphaeroides*, the Co^2+^ concentration required in the growth medium (M27) is about 1 μM. However, high concentrations of cobalt result in toxic effects that can decrease its growth: the effective concentration (EC_50_) of Co^2+^ to reduce the growth rate of *R. sphaeroides* by 50% of the value observed under control conditions is 0.8 mM (Giotta et al., 2006). In various studies with *R. sphaeroides* that focused on the effects of cobalt stress, the growth medium had about 5 mM Co^2+^ concentration (Pisani et al., 2009; Losurdo et al., 2010; Belviso et al., 2013; Volpicella et al., 2014), as *R. sphaeroides* could still have a consistent biomass yield, even at about 5 mM CoCl_2_ (Giotta et al., 2006). The high cobalt tolerance of *R. sphaeroides* has led to further investigations of the mechanisms of cobalt response and resistance in this species. Italiano et al. (2009) studied the bioadsorption and bioaccumulation of Co, Ni and Mg, using inductively coupled plasma atomic emission spectroscopy and attenuated total reflection Fourier transform infrared spectroscopy (ATR-FTIR). Their results revealed the competition of Co^2+^ with Mg^2+^ for the same carboxylate binding groups on the outer membrane of *R. sphaeroides* (Italiano et al., 2009). In addition, X-ray absorption spectroscopy results revealed extensive binding of cobalt ions to sulfolipids on the photosynthetic membrane of *R. sphaeroides* (Belviso et al., 2013). Lipidome analysis of *R. sphaeroides* in the presence of cobalt and chromate ions stress using thin-layer chromatography coupled with matrix-assisted laser desorption ionization mass spectrometry (MALDI-MS) showed that, upon Co^2+^ ion stress, a significant increase occurred in sulfoquinovosyldiacylglycerols and a decrease in phosphatidylglycerols was observed in the membrane lipids of *R. sphaeroides* (Calvano et al., 2013). The photosynthetic membrane proteome analysis of *R. sphaeroides* revealed that many proteins of the photosynthetic apparatus were differentially expressed upon exposure to cobalt stress (Italiano et al., 2011).

Despite the biotechnological importance of both *R. sphaeroides* and cobalt, there is a limited number of studies that investigated the genetic factors associated with cobalt resistance in *R. sphaeroides*: Volpicella et al. (2014) selected cobalt-sensitive mutants from a *R. sphaeroides* transposon insertion library and performed comparative transcriptomic and genomic analyses of a selected cobalt-sensitive mutant. The transcriptome profiling results showed that an ATP-binding cassette (ABC) sugar transporter was significantly downregulated in the cobalt-sensitive mutant strain which had a mutation (transposon insertion) in the RSP_7363 gene that encodes a hypothetical protein. Interestingly, the mutant strain was not significantly inhibited in the presence of high levels of cobalt stress when cultured under photosynthetic conditions. Thus, it was concluded that an additional energetic resource provided by the ABC sugar transporter may be required to overcome the cobalt stress, and the ability of the mutant strain to exploit light energy during photosynthetic conditions may compensate for the impairment of the ABC sugar transporter (Volpicella et al., 2014). More recently, using a bioinformatics approach, functional annotation of heavy metal tolerance genes of *R. sphaeroides* were analyzed and the majority of those genes (255 genes, about 63%) were encoding metal-dependent enzymes or enzymes reducing metallic compounds to elemental metals. In addition, the second largest group (127 genes, about 34%) was encoding transporters that involve metal-binding proteins and ATPase translocases, making *R. sphaeroides* a good model organism for studying heavy metal tolerance and for bioremediation applications (Johnson et al., 2019). The exact molecular mechanisms of metal or cobalt-resistance in *R. sphaeroides* are yet to be clarified.

Evolutionary engineering or adaptive laboratory evolution (ALE) is a powerful strategy to improve industrially important microbial properties with an unknown molecular basis (Mavrommati et al., 2023; Topaloğlu et al., 2023). It exploits nature’s evolutionary principles based on random mutation and systematic selection to favor a desirable phenotype (Butler et al., 1996; Sauer, 2001). In our research group, we have successfully applied evolutionary engineering to obtain cobalt-resistant (Çakar et al., 2009; Alkım et al., 2013), iron-resistant (Balaban et al., 2020), nickel-resistant (Küçükgöze et al., 2013), silver-resistant (Terzioğlu et al., 2020) and oxidative stress-resistant (Kocaefe-Özşen et al., 2022) yeast (*S. cerevisiae*) strains. In addition, we have also successfully applied evolutionary engineering to industrially important bacteria such as *Rhodobacter capsulatus* (Gökçe et al., 2012, 2017), and *Bacillus boroniphilus* (Şen et al., 2011), to improve their heat- and boron-resistance, respectively. Using comparative genomic, transcriptomic, and/or physiological analysis of the highly-resistant evolved and original/reference strains, the complex molecular basis of the desired properties, such as resistance to a particular stress type, can be identified.

The aim of this study was to obtain a cobalt-hyperresistant *R. sphaeroides* strain by using evolutionary engineering and to investigate the molecular basis of cobalt resistance in the metabolically diverse bacterium *R. sphaeroides* by comparative genomic and physiological analyses. For this purpose, *R. sphaeroides* reference strain was subjected to an evolutionary batch selection procedure at gradually increased cobalt concentrations between 0.1–15 mM CoCl_2_ in the culture medium. Individual colonies (mutants) were randomly picked upon growing the final population of selection on solid plates. The colonies were then physiologically characterized based on their stress resistance and genetic stability, to identify an evolved strain (G7) with superior resistance properties. Whole-genome re-sequencing of the evolved strain G7 and the reference strain revealed single nucleotide polymorphisms (SNPs) that may be associated with the high cobalt-resistance of the evolved strain. The resistance tests against cobalt and other stressors, growth physiological analyses and genome sequence data showed that the growth of the evolved strain was not inhibited by 4 mM CoCl_2_ that significantly inhibited the reference strain’s growth and higher amounts of cobalt ions were accumulated in or on the evolved strain, compared to the reference strain, indicating a bioremediation potential for the evolved strain. The SNPs were observed in diverse genes of the evolved strain related to transcriptional regulation, NifB family-FeMo cofactor biosynthesis, putative virulence factors, TRAP-T family transporter, sodium/proton antiporter, and many genes with unknown function that may play a key role in the cobalt resistance of *R. sphaeroides*.

## 2 Materials and methods

### 2.1 Strain, media and growth conditions

The *Rhodobacter sphaeroides* R-26 strain (Dr. Massimo Trotta’s laboratory collection) was used in this study. The original carotenoidless strain is known to have a tendency to revert (Kwa et al., 2008). In this study, it was allowed to revert to the reddish form to avoid any further stress imposed by the maintenance of the mutant. For this purpose, the chemoheterotrophically grown R-26 culture was directly placed under strict anaerobic photosynthetic conditions, as described previously (Kiley et al., 1988) which has been shown to increase the frequency of revertants in the population (Kiley et al., 1988). The reverted strain - named RR-26 - was used as the reference strain (RS) throughout this study and is now available from the laboratories. Unless otherwise stated, *R. sphaeroides* cultures in this study were grown under aerobic conditions in the dark, at 37°C and 150 rpm using Medium 27 (M27) of Deutsche Sammlung von Mikroorganismen und Zellkulturen GmbH (DSMZ). M27 consisted of 100 μg/L ZnSO_4_.7H_2_O, 30 μg/L MnCl_2_.4H_2_O, 300 μg/L H_3_BO_3_, 200 μg/L CoCl_2_.6H_2_O, 10 μg/L CuCl_2_.2H_2_O, 20 μg/L NiCl_2_.6H_2_O, and 30 μg/L Na_2_MoO_4_.2H_2_O as micronutrients; and 500 mg/L KH_2_PO_4_, 800 mg/L MgSO_4_.7H_2_O, 400 mg/L NaCl, 400 mg/L NH_4_Cl, 50 mg/L CaCl_2_.2H_2_O, 1,500 mg/L D-L malic acid, 2000 mg/L yeast extract, 1 mg/L p-amino benzoic acid, and 18 mg/L iron (II) citrate as macronutrients that were dissolved in ddH_2_O (Italiano et al., 2009). The pH of the medium was adjusted to 6.8 after macronutrients were dissolved in deionized water and autoclaved at 121°C for 15 min. Micronutrients were filter-sterilized using 0.22 μm pore-size filters, and then added into the sterilized M27 medium. The medium was stored at 4°C in the dark. For solid plate cultivations, Luria-Bertani (LB) medium was used. It included 10 g NaCl, 10 g tryptone, 5 g yeast extract and 20 g agar in 1 L of ddH_2_O (Bertani, 1951). Growth was monitored by optical density measurements at 660 nm (OD_660_), using a spectrophotometer (Shimadzu UV- 1700, Japan). Cultures were stored at −80°C in 2 mL M27 medium containing 30% (v/v) glycerol.

### 2.2 Evolutionary engineering of cobalt-resistant *Rhodobacter sphaeroides* strains

To determine the initial cobalt stress levels to be applied in evolutionary engineering experiments, overnight pre-culture of the reference strain grown aerobically in the dark in M27 medium at 37°C and 150 rpm was inoculated into 15 mL fresh M27 medium containing 0–5 mM CoCl_2_ in 50 mL culture tubes, to an initial OD_660_ of 0.1. Cultures were then incubated aerobically at 37°C, 150 rpm in the dark for 24 h, serially diluted and spread on LB plates. Three independent values of colony-forming units (CFU) were determined by direct plate counting, upon 72-h incubation at 37°C. The survival rates under various cobalt stress conditions were calculated by dividing the CFU values under cobalt stress condition to the CFU value of the control culture. Successive batch selection was used as an evolutionary engineering strategy by gradually increasing CoCl_2_ concentration throughout the passages or populations. For this purpose, 100 μL of the reference strain’s (RS) stock culture at - 80°C were inoculated into 15 mL fresh M27 medium in a 50 mL culture tube, and incubated overnight at 37°C and 150 rpm, aerobically in the dark. This pre-culture was then inoculated into 15 mL fresh M27 medium containing 0.1 mM CoCl_2_ in a 50 mL culture tube, and incubated overnight again aerobically at 37°C in the dark and at 150 rpm. This culture treated with 0.1 mM CoCl_2_ stress was named as the first population of selection. This procedure was repeated for a total of 64 passages or populations, by gradually increasing the CoCl_2_ concentration at each passage. For each population, the cultures were also incubated under control conditions in M27 medium, in the absence of CoCl_2_. These control cultures were used to determine the survival rates for each population, by dividing the final OD_660_ value of the stressed culture to that of the control culture. The final population of the selection was diluted by 10^6^ –fold, and spread on solid LB plates to randomly pick individual mutant colonies after 4 days of incubation at 37°C.

### 2.3 Estimation of stress resistance

Cobalt resistance levels of selected individual mutants from the final population were quantitatively determined using the Most Probable Number (MPN) method (Russek and Colwell, 1983; Çakar et al., 2005), by applying serial dilutions in 96-well plates containing 180 μL M27 (control), and varying amounts of CoCl_2_ in 180 μL M27 (cobalt stress condition). The MPN method was applied as five replicates. Briefly, in control plates, cultures were diluted in the range of 10^−1^ to 10^−12^, and in plates with 2 mM, 4 mM, 6 mM, 8 mM or 15 mM CoCl_2_ stress, cultures were diluted in the 10^−1^ to 10^−8^ range. After incubation at 37°C for 4 days, the number of surviving cells at different cobalt concentrations was estimated by using MPN tables (Lindquist, 2020).

Resistance of selected individual mutants to CoCl_2_ and other stressors were determined using spot assay. Individual mutants and the RS were revived in M27 medium. During exponential growth phase of the cells, cultures were inoculated into 10 mL M27 in 50 mL culture tubes at a starting OD_660_ of 0.2 and incubated aerobically at 37°C, 150 rpm in the dark. When the cultures reached an OD_660_ of 1.2, 5 OD_660_ units/mL cells were collected by centrifugation at 10,000 × g for 5 min. Pellets were resuspended in 1 mL M27 and serial dilutions of the cultures were made and spotted on solid LB plates containing 1–5 mM CoCl_2_ as cobalt stress conditions, and on an LB plate without CoCl_2,_ as the control condition. Similarly, to test for cross-resistance to other stressors, serial dilutions of the cultures were spotted on solid LB plates containing ethanol (8% v/v), NaCl (0.5 M), AlCl_3_ (5 mM), CuCl_2_ (0.5 mM), NiCl_2_ (2.0–2.4 mM), MgCl_2_ (0.5 - 1 M), H_2_O_2_ (1–2 mM), FeCl_2_ (4–5 mM), MnCl_2_ (10 mM), H_3_BO_3_ (30–50 mM), caffeine (20 mM), (NH_4_)_2_ Fe (SO_4_)_2_ (5 mM), and ZnCl_2_ (1 mM). Plates were incubated at 37°C for 4 days.

### 2.4 Growth analyses

Growth profiles of RS and a cobalt-resistant, evolved strain were obtained spectrophotometrically at OD_660_ and by dry weight measurements. For this purpose, firstly, precultures were prepared by inoculating 100 μL of stock cultures into 10 mL fresh M27 medium in 50 mL culture tubes. After overnight incubation at 37°C and 150 rpm aerobically and in the dark, cultures were inoculated into 500 mL flasks containing 100 mL M27 (control condition) and 100 mL M27 with 4 mM CoCl2 (cobalt stress condition), to an initial OD_660_ value of 0.2, and grown again aerobically in the dark, at 37°C and 150 rpm. Samples were taken from the cultures at two-hour intervals. For cell dry weight analysis, 1.5 mL microfuge tubes were dried in an oven at 80°C for 48 h, placed in a desiccator for 3 h, and then weighed. Two mL of samples were withdrawn from the cultures, transferred to the pre-weighed microfuge tubes, and centrifuged at 15,000 g for 5 min. The culture supernatants were discarded and pellets were placed in an oven at 80°C to dry. After 48 h, the tubes were reweighed. The experiments were performed in triplicate.

### 2.5 Determination of cellular cobalt contents by flame atomic absorption spectrometry (FAAS)

Pre-cultures were prepared by inoculating 100 μL of the stock cultures into 10 mL fresh M27 in 50 mL culture tubes, and incubating overnight and aerobically in the dark at 37°C and 150 rpm. After overnight incubation, pre-cultures were inoculated into 10 mL fresh M27 (control condition), and M27 with 4 mM CoCl_2_ (cobalt stress condition) in 50 mL culture tubes, to an initial OD_660_ of 0.25. Cultures were incubated under aerobic conditions in the dark at 37°C, 150 rpm for 24 h. They were then centrifuged at 5000 g for 15 min. The supernatants were discarded and the cell pellets were washed twice by using distilled water, dried at 110°C for 2 h and the cell dry weights were measured as described in the previous section. Cell pellets were then hydrolyzed at 90°C for 2 h, by using 10 M HNO_3_. Cobalt contents were determined by using a Flame Atomic Absorption Spectrophotometer (FAAS), an Analytik-Jena Model AAS Vario 6 (Germany), with a hollow cathode lamp. The wavelength and slit width values were 240.7 and 0.2 nm. The experiments were performed in triplicate.

### 2.6 Whole genome re-sequencing

For comparative whole genome re-sequencing, 100 μL of the stock cultures of the RS and the evolved strain were inoculated into 10-mL fresh M27 medium in 50 mL culture tubes. Upon overnight incubation under aerobic conditions at 37°C, and 150 rpm in the dark, 500 μL of cultures were centrifuged at 15000 g for 5 min and DNA isolation was performed by using the MasterPure DNA Purification Kit (Epicentre) according to the manufacturer’s protocol. DNA purity and concentration were determined by using a NanoDrop 2000 UV–Vis spectrophotometer (Thermo Fisher Scientific). The sequencing libraries were prepared by Ion Xpress Plus Fragment Library Kit (Thermo Fisher Scientific) and Ion 540Chip Kit, following the manufacturer’s protocol. Next generation sequencing was performed on the Ion S5 Platform (Thermo Fisher Scientific), by using automated library prep platform Ion ChefTM (Thermo Fisher Scientific). The quality control of raw data was performed by using FastQC software v.0.11.9 (Babraham Bioinformatics). The Trimmomatic v.0.39 software (Bolger et al., 2014) was used to remove adapter sequences and low-quality sequences from reads. The reference genome of *R. sphaeroides* 2.4.1 was used to align the reads from the cobalt-resistant, evolved strain and the *R. sphaeroides* R-26 reference strain used in this study. Subsequently, the variations shared between the evolved strain and R-26 were identified. Once these common variations were defined, the remaining ones were determined to be specific to the evolved strain. Burrows– Wheeler aligner MEM v.0.7.1 (Li and Durbin, 2009) was used as a sequence alignment tool. Variant calling was carried out by using Genome Analysis Toolkit (GATK) v.3.8.0 (DePristo et al., 2011) and each nucleotide change was inspected with Genome Browse v2.1.2 (GoldenHelix). In-house R scripts were used to eliminate low-quality single nucleotide polymorphisms (SNPs). Remaining SNPs were annotated by using Variant Effect Predictor v.90. Whole-genome re-sequencing data have been deposited in the NCBI Sequence Read Archive (SRA) under BioProject PRJNA1047122.

## 3 Results

### 3.1 Evolutionary engineering of cobalt-resistant *Rhodobacter sphaeroides*

Before starting with the evolutionary engineering experiments, the reference strain was cultivated in the presence of varying levels of cobalt stress (0–5 mM CoCl_2_), and the survival rates of the cultures were determined by direct plate counting. According to the survival rate values of these cultures, 0.1 mM CoCl_2_ was chosen as the initial, mild cobalt stress level for evolutionary selection experiments (Supplementary Figure S1). The reference strain could not grow at CoCl_2_ concentrations higher than 10 mM (data not shown).

As the evolutionary engineering strategy, successive batch selection was used by gradually increasing CoCl_2_ concentration from an initial level of 0.1 mM up to 15 mM for 64 passages or populations. The CoCl_2_ concentration was increased by 0.05 mM at each population between the first and 13^th^ populations (between 0.1 mM and 0.7 mM CoCl_2_), and then gradually increased at varying steps, as indicated in Table 1, reaching the final cobalt stress level of 15.0 mM CoCl_2_ at the 64^th^ passage of selection. The 64^th^ passage of the selection was named as the final population of selection, and was spread on LB plates. Upon incubation for 4 days, eight individual colonies were randomly picked from the final population plates as individual mutant strains to investigate their stress resistance. An evolved strain was defined as a mutant strain that has been isolated as a colony from the final (64^th^) population of selection.

**Table 1:** CoCl_2_ stress levels that were applied during 64 successive batch populations of evolutionary engineering selection.

Population number	CoCl_2_ stress level increase (mM) at each population	CoCl_2_ stress level (mM) of the population
1–13	0.05	0.1–0.7
13–16	0.1	0.7–1.0
16–49	0.2	1.0–7.6
49–50	0.4	7.6–8.0
50–64	0.5	8.0–15.0

### 3.2 Stress resistance properties of the evolved strains

**Figure 1:**
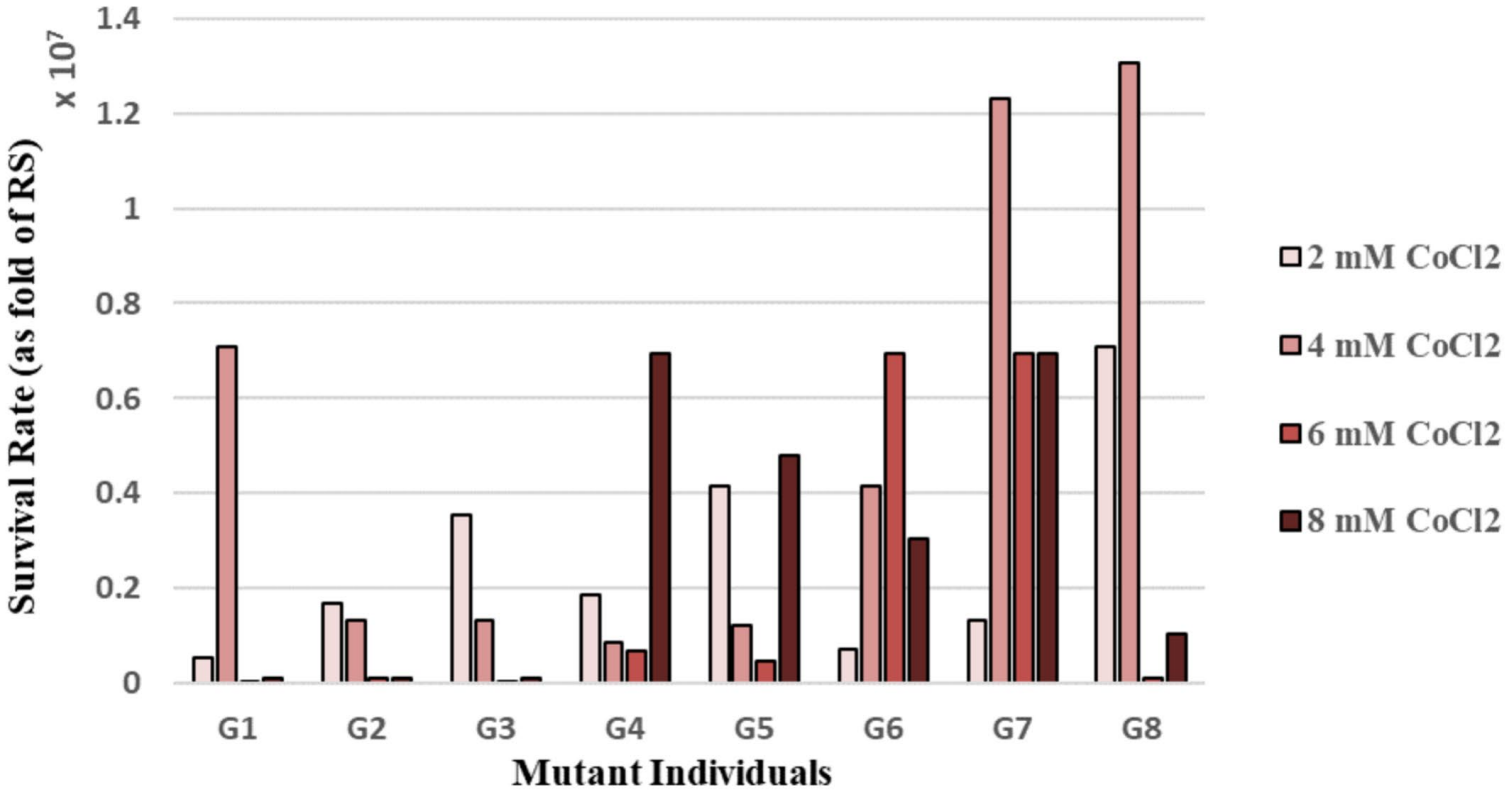
Survival rates [as fold of the reference strain (RS)] of the mutant individual colonies of the final population of selection, upon continuous exposure to varying levels of cobalt stress, as determined by the Most Probable Number (MPN) method.

The eight individual mutant colonies (named G1-G8) randomly picked from the final population plate had varying red pigmentation intensities: G1, G2 and G3 had pink color, G4 and G5 were red, and G6, G7 and G8 were dark red. The quantitative estimation of cobalt resistance of the evolved strains was made by using the MPN method. For this purpose, the individual mutants and RS were cultivated in 96-well plates in the absence and presence of 2–8 mM CoCl_2_ stress. These CoCl_2_ concentrations were chosen based on the literature information (Giotta et al., 2006; Volpicella et al., 2014) and our cobalt stress survival data for the reference strain determined by direct plate counting (Supplementary Figure S1). The stress survival rates of the individual mutants were calculated by dividing the estimated number of the individual mutant cells upon CoCl_2_ stress exposure to that under control (no stress) condition. The stress survival rates of the individual mutants were normalized to that of the RS. The results revealed that the individual mutants showed significantly higher resistance to CoCl_2_ stress than the RS. Among them, the mutant G7 had higher cobalt resistance levels than most of the other mutant individuals at 4–6 and 8 mM CoCl_2_ (Figure 1).

**Figure 2:**
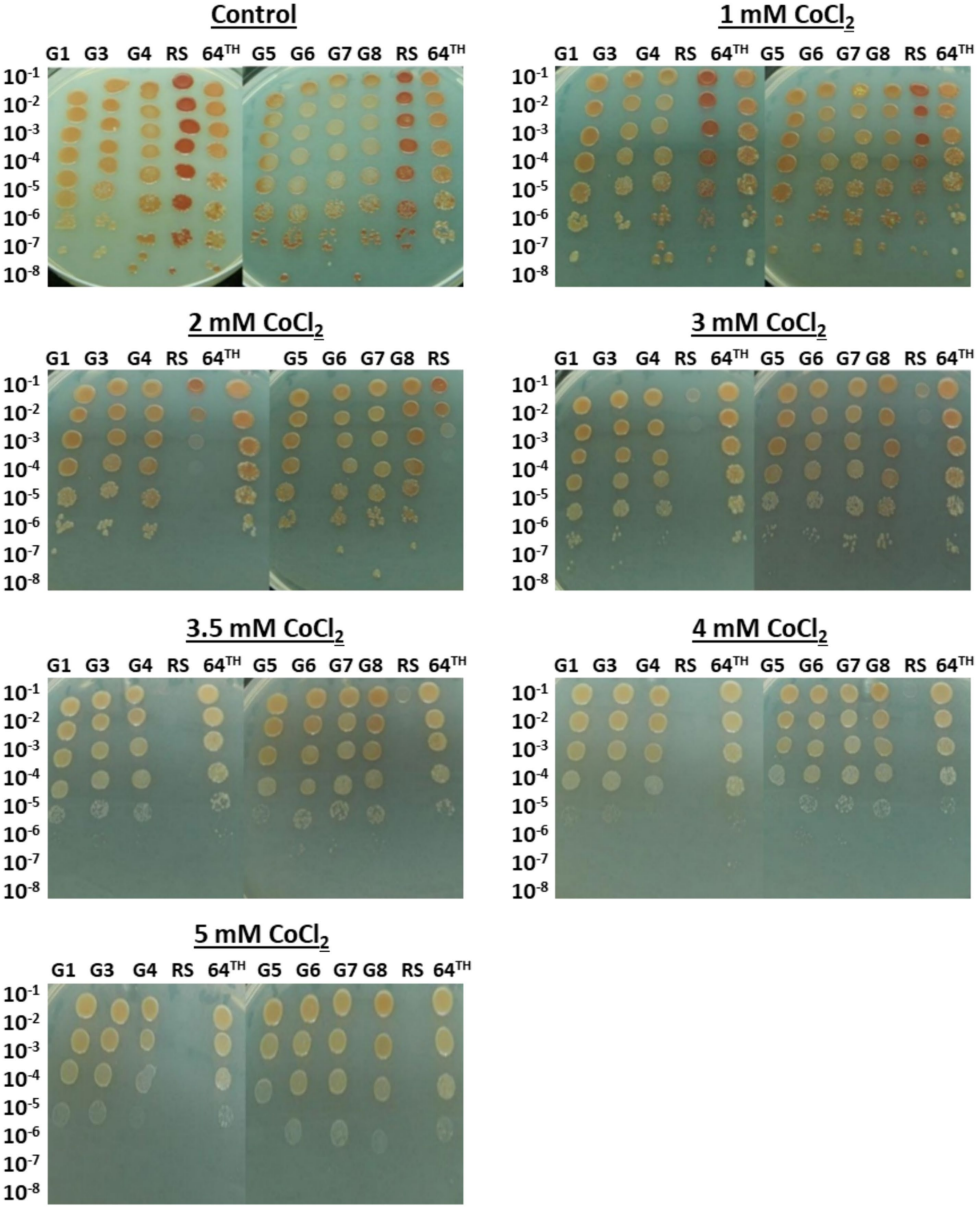
Cobalt stress resistance of the mutant individual colonies, final (64th) population of selection and the reference strain (RS), as determined by the spot assay. The cultures were spotted on LB (control condition) and 1–5 mM CoCl_2_-containing LB (stress conditions) plates in serial dilutions, and incubated at 37°C for 4 days.

The spot assay results of the evolved strains confirmed the CoCl_2_ stress resistance of the evolved strains. The cobalt stress resistance levels of the evolved strains G1-G8 and the 64^th^ population were determined by spotting serial dilutions of the cultures on LB plates containing 0–5 mM CoCl_2_. These CoCl_2_ concentrations were also chosen based on the literature information (Giotta et al., 2006; Volpicella et al., 2014) and our cobalt stress survival data for the reference strain determined by direct plate counting (Supplementary Figure S1). As the individual mutant G2 became contaminated, it was excluded from the spot assay and further analyses. The spot assay results revealed that the evolved strains had significantly higher resistance than the RS. At CoCl_2_ concentrations higher than 3 mM on LB plates, the growth of the RS was completely inhibited. However, the evolved strains could grow up to higher dilution levels at these inhibitory CoCl_2_ concentrations which indicates their high cobalt resistance (Figure 2).

### 3.3 Cross-resistance of cobalt-resistant mutant individuals to other stress factors

**Figure 3:**
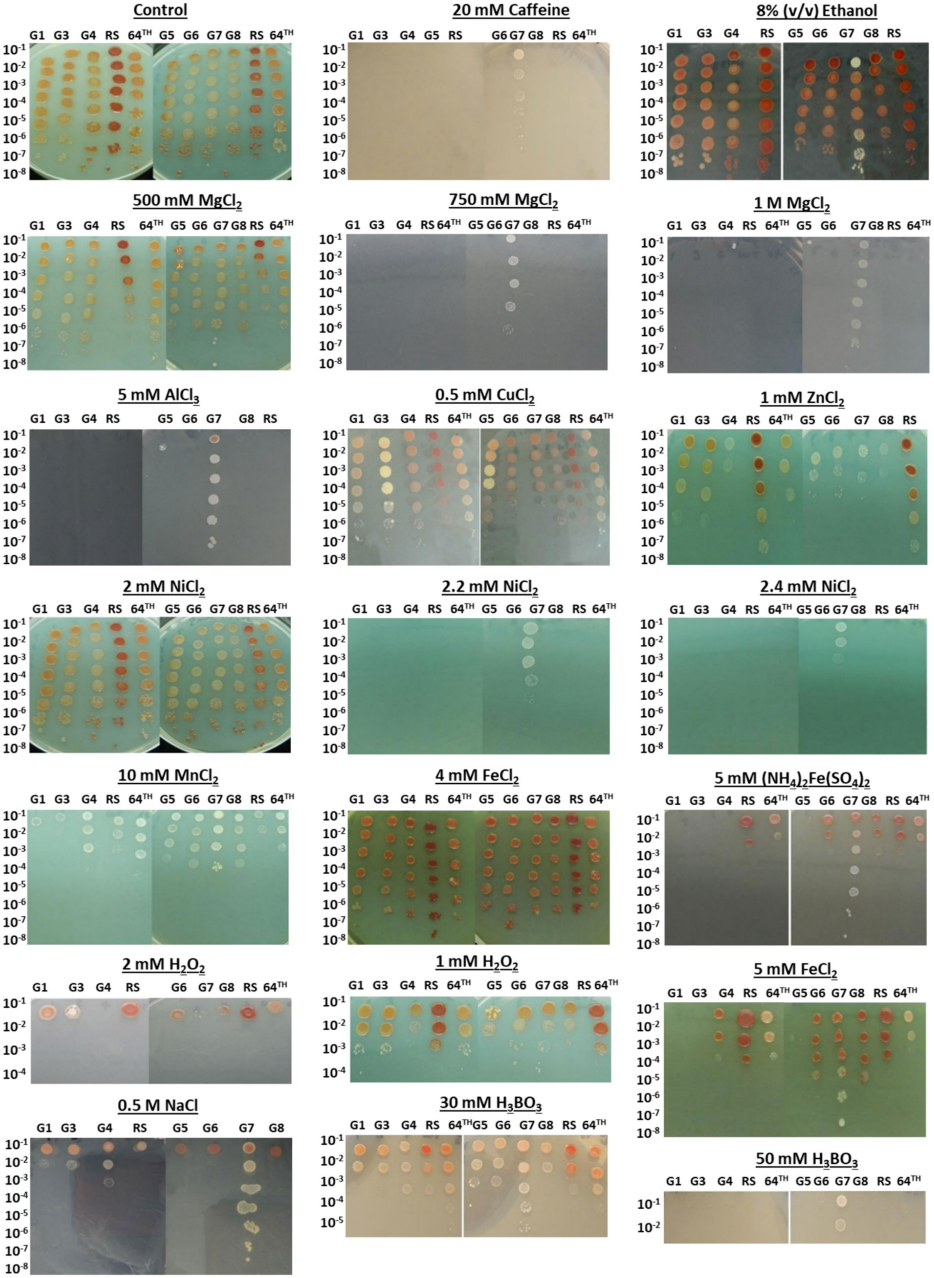
Cross- resistance of the mutant individual colonies, final (64th) population of selection and the reference strain (RS) against various stressors, as determined by the spot assay. The cultures were spotted on LB plates (control condition) and LB plates with various stressors (stress conditions) in serial dilutions, and incubated at 37°C for 4 days.

It is known that bacterial cultures can develop cross-resistance against other stress types, when they are exposed to one stress type. This phenomenon is also known as environmental adaptation or cross-protection (Rangel, 2011). To test for the potential cross-resistance of the cobalt-resistant evolved strains against other stressors, spot assay was performed in the presence of various stressors, as described in the Materials and Methods section. The concentrations of other stressors were determined based on our previous cross-resistance studies (Çakar et al., 2009; Terzioğlu et al., 2020; Kocaefe-Özşen et al., 2022) and preliminary optimization experiments to find those stressor concentrations that can cause a clear growth difference between the reference strain and the evolved strains in spot assays. For NaCl, for example, the required amount provided in the M27 medium for the growth of *R. sphaeroides* is about 6.6 mM (400 mg/L). Although it has been reported that NaCl concentrations up to 300 mM did not cause a significant growth inhibition in *R. sphaeroides*, complete growth inhibition was observed at NaCl concentrations higher than 1 M (Labarile et al., 2021). Thus, 0.5 M NaCl was chosen as the NaCl stress condition in this study, to test for the potential halotolerance of the evolved strains. The results revealed that the evolved strain G7 was highly cross-resistant to most of the stressors tested. Among all evolved strains and the RS, it was the only strain that could grow in the presence of 20 mM caffeine, 2.2 and 2.4 mM NiCl_2_, 0.75 and 1 M MgCl_2_, 50 mM H_3_BO_3_ and 5 mM (NH_4_)_2_Fe(SO_4_)_2_ stress. In addition, G7 also showed significantly higher resistance than the reference strain and the other evolved strains against 0.5 M NaCl, 5 mM AlCl_3_ and 5 mM FeCl_2_ stresses (Figure 3).

As the evolved strain G7 was highly resistant to cobalt stress and showed high levels of cross-resistance against many other stressors, this robust strain was chosen for genetic stability test and further detailed investigations. The genetic stability test was performed by growing G7 in M27 medium in the absence of cobalt stress for 10 successive passages and testing for the cobalt resistance of each passage by growth on LB plates containing 4 mM CoCl_2_. The results revealed that there was no loss of resistance levels upon this successive cycle (data not shown).

### 3.4 Growth profiles of the RS and the evolved strain G7

**Figure 4:**
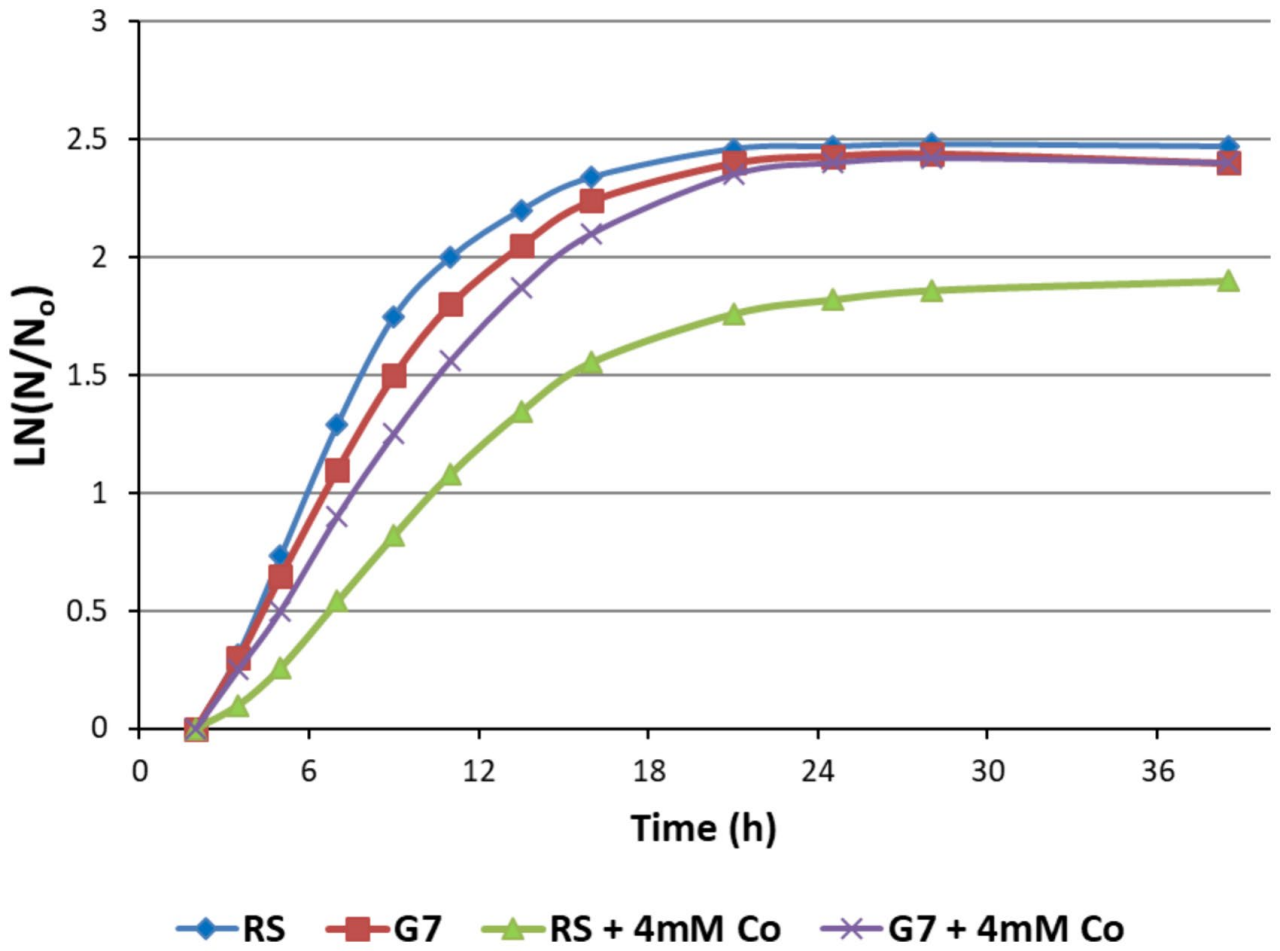
Growth curves of the reference strain (RS) and the cobalt-resistant, evolved strain G7 represented as ln (N/N_0_), in the presence and absence of 4 mM CoCl_2_. N is the number of cells and N_0_ is the initial number of cells in the culture. Cultures were grown at 37°C for 38 h, using M27 medium.

The growth behavior of the RS and the evolved strain G7 was investigated in M27 medium under aerobic conditions in the dark, both in the presence and absence of cobalt stress (4 mM CoCl_2_). The results revealed that 4 mM CoCl_2_ stress had no significant inhibitory effect on the evolved strain G7, although it significantly inhibited the growth of the RS (Figure 4).

**Figure 5:**
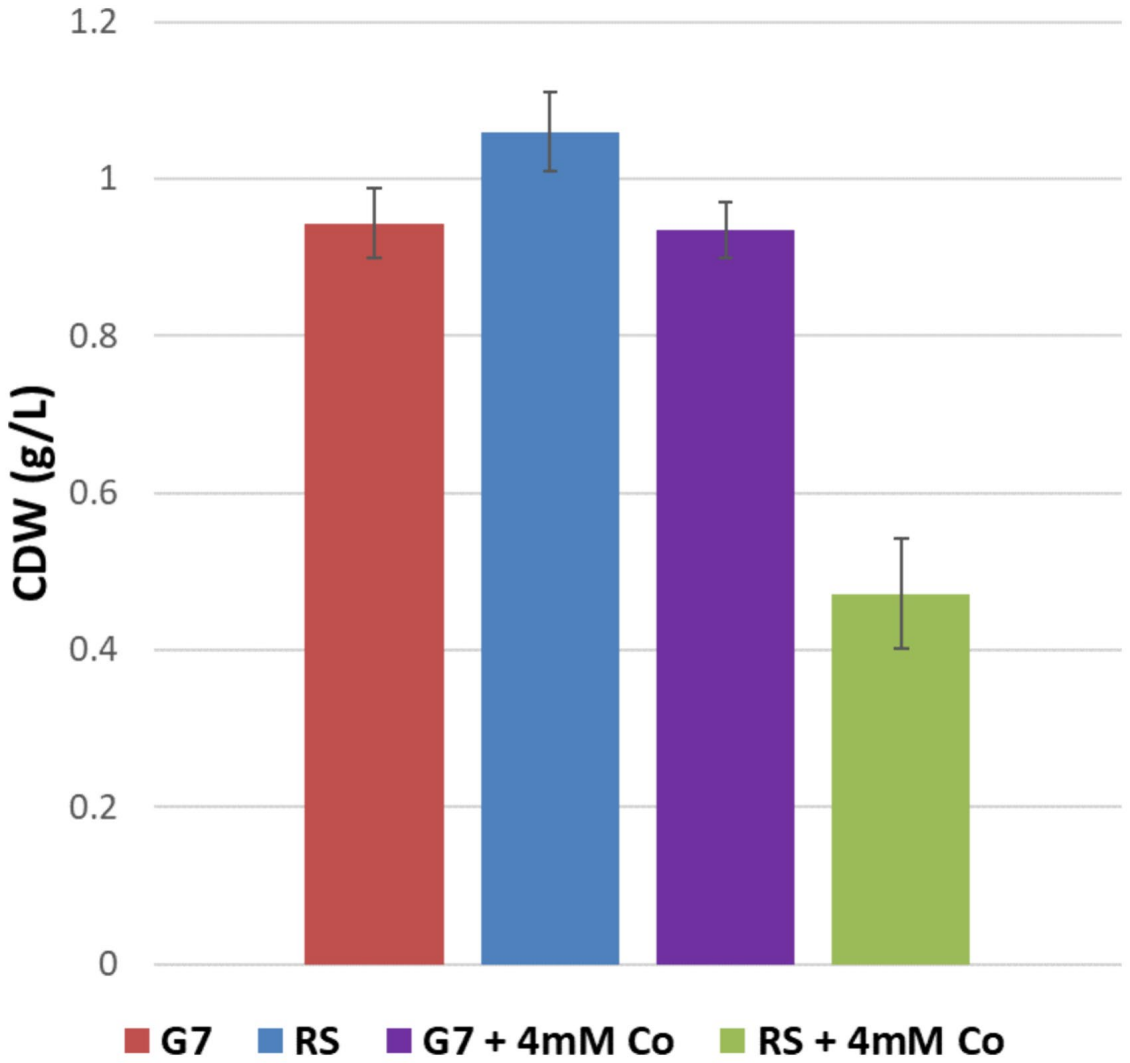
Final cell dry weights (CDW) of the reference strain (RS) and the cobalt-resistant, evolved strain G7, in the presence and absence of 4 mM CoCl_2_. Cultures were grown in M27 medium at 37°C for 38 h.

The maximum specific growth rates of G7 in the absence and presence of 4 mM CoCl_2_ stress were calculated as 0.19 h^−1^ and 0.13 h^−1^, respectively. For the RS, however, the μ_max_ values in the absence and presence of 4 mM CoCl_2_ stress were 0.23 h^−1^, and 0.07 h^−1^, respectively. The strong inhibitory effect of 4 mM CoCl_2_ stress on the RS and the strong cobalt resistance of G7 were also verified by the final cell dry weights (CDW) of the cultures (Figure 5).

### 3.5 Cobalt contents of the RS and the evolved strain G7

The cobalt contents of the RS and the evolved strain were determined using Flame Atomic Absorption Spectrometry (FAAS). Both strains were grown aerobically, in the dark, and in the presence of 4 mM CoCl_2_ which is the same cobalt stress level used for growth experiments, to enable comparison of growth profiles and cobalt content data under the same stress conditions. The results revealed that the cobalt-resistant, evolved strain G7 had a significantly higher cobalt content (about 13 mg/g CDW) than the RS which accumulated only about 0.6 mg cobalt/g CDW (Table 2).

**Table 2:** Cobalt contents [in mg/g cell dry weight (CDW)] of G7 and RS in the presence of 4 mM CoCl_2_ stress.

Stress condition	Replicate no	RS (mg/g CDW)	G7 (mg/g CDW)
	1	0.60	8.64
4 mM CoCl_2_	2	0.69	15.84
	3	0.41	13.98
Average		**0.57 ± 0.14**	**12.82 ± 3.74**

### 3.6 Comparative whole-genome resequencing of the evolved strain G7

Whole-genome resequencing results revealed 23 mutations in the evolved strain G7 that were not found in the RS. One start-loss, 14 non-synonymous (missense) and 8 synonymous mutations were identified in the G7 genome (Table 3).

**Table 3:** Mutations that were identified in the cobalt-resistant, evolved *R. sphaeroides* strain G7, based on comparative whole genome-resequencing of G7 and the reference strain (RS).

ORF ID	Gene name	Genomic location	Type of mutation	Amino acid change	Codon change	Description (bacteria.ensembl.org)
RSP_7501	–	1:140292	Start lost	M/T	aTg/aCg	Hypothetical protein
RSP_1810	mviN	1:397063	Missense variant	V/A	gTc/gCc	Putative virulence factor, MviN
RSP_1810	mviN	1:397064	Missense variant	V/I	Gtc/Atc	Putative virulence factor, MviN
RSP_2320	–	1:943423	Missense variant	Y/H	Tac/Cac	TRAP-T family transporter, periplasmic binding protein
RSP_2417	–	1:1049374	Missense variant	E/K	Gag/Aag	Transglutaminase-like enzyme, predicted cysteine protease
RSP_2932	hutC	1:1610205	Missense variant	V/A	gTg/gCg	Histidine utilization repressor, gntR family
RSP_0395	rpoD	1:2126519	Missense variant	C/R	Tgc/Cgc	RNA polymerase, sigma 70 subunit, RpoD
RSP_0546	nifB	1:2282436	Missense variant	A/T	Gcg/Acg	NifB family--FeMo cofactor biosynthesis protein
RSP_0850	mbfA	1:2600637	Missense variant	A/P	Gct/Cct	Membrane-bound ferritin
RSP_0998	nhaD	1:2757127	Missense variant	S/G	Agt/Ggt	Sodium/proton antiporter, NhaD family
RSP_3478	–	2:548776	Missense variant	G/D	gGc/gAc	Type VI secretion protein, EvpB/VC_A0108 family
RSP_3791	–	2:897168	Synonymous variant	A	gcC/gcG	Hypothetical protein
RSP_3791	–	2:897177	Synonymous variant	G	ggG/ggC	Hypothetical protein
RSP_3791	–	2:897225	Synonymous variant	G	ggT/ggC	Hypothetical protein
RSP_3791	–	2:897227	Missense variant	G/S	Ggt/Agt	Hypothetical protein
RSP_3791	–	2:897243	Synonymous variant	P	ccC/ccT	Hypothetical protein
RSP_3791	-	2:897264	Synonymous variant	A	gcC/gcG	Hypothetical protein
RSP_3791	-	2:897297	Synonymous variant	L	ctG/ctC	Hypothetical protein
RSP_3791	-	2:897313	Missense variant	S/F	tCc/tTc	Hypothetical protein
RSP_3791	-	2:897321	Synonymous variant	V	gtC/gtG	Hypothetical protein
RSP_3791	-	2:897329	Synonymous variant	R	Agg/Cgg	Hypothetical protein
RSP_3988	-	B:63268	Missense variant	V/A	gTg/gCg	Putative glycosyltransferase
RSP_4277	-	E:22638	Missense variant	A/V	gCg/gTg	Transcriptional regulator, MerR family

The start-loss mutation was found in the open reading frame (ORF) ID: RSP_7501 that encodes a hypothetical protein. Two missense mutations were identified in the MviN gene that codes for the putative virulence factor MviN. Other missense mutations were identified in RSP_2320 that encodes a TRAP-T family transporter, periplasmic binding protein; in the hutC gene encoding a histidine utilization repressor of the gntR family; rpoD gene that encodes the RNA polymerase sigma 70 subunit RpoD; nifB gene coding for an FeMo cofactor biosynthesis protein of the NifB family; mbfA gene that codes for a membrane-bound ferritin; nhaD gene encoding a sodium/proton antiporter of the NhaD family; RSP_2417 that codes for a transglutaminase-like enzyme, a predicted cysteine protease; RSP_3478 coding for a type VI secretion protein; RSP_3988 encoding a putative glycosyltransferase; and RSP_4277 encoding a transcriptional regulator of MerR family. Interestingly, two missense and eight synonymous mutations were observed in RSP_3791 that encodes a hypothetical protein (Table 3).

## 4 Discussion

In this study, we have successfully obtained a highly cobalt-resistant and genetically stable *R. sphaeroides* strain by evolutionary engineering. The evolved strain was isolated from the final (64^th^) population of the successive batch selection in the presence of increasing levels of CoCl_2_ stress, starting from 0.1 mM CoCl_2_ in the first population and gradually increasing up to 15 mM in the final population. To our knowledge, 15 mM CoCl_2_ is the highest CoCl_2_ stress level that has been reported so far, at which *R. sphaeroides* cells could survive.

Our physiological analyses of the highly cobalt-resistant, evolved strain G7 revealed that, in addition to its high level of cobalt resistance, G7 was highly cross-resistant against many other stressors. These stressors include FeCl_2_, (NH_4_)_2_Fe(SO_4_)_2_, NaCl, MgCl_2_, H_3_BO_3_, AlCl_3_, NiCl_2_, and caffeine (Figure 3). With its dark red color, the highly cobalt-resistant, evolved strain G7 had also a high pigmentation intensity. The relationship between stress conditions and carotenoid production has been reported previously for diverse organisms. The mollusc *Perna viridis* with high carotenoid content was shown to be highly resistant to environmental pollution, and the concentration of carotenoids was high when the heavy metal concentrations including cobalt were high in the tissues of *P. viridis* (Tewari et al., 2001). In the copper-tolerant yeast strain *Rhodotorula mucilaginosa* RCL-11, the presence of copper and the oxidative stress agent H_2_O_2_ in the culture medium increased its carotenoid pigment levels and changed the carotenoid profiles (Irazusta et al., 2013). Moreover, in photosynthetic bacteria (*Rhodopseudomonas*) that could tolerate high salinity, it was shown that salinity stress of about 50 g/L NaCl enhanced carotenoid and bacteriochlorophyll production (Wang et al., 2017). Thus, the high level of pigmentation in our evolved strain G7 may be associated with its improved tolerance against various stressors.

Iron is an essential element for living organisms, as it is involved as a cofactor in several enzymes and regulatory proteins. Studies with *Escherichia coli* and *Salmonella enterica* identified iron–sulfur [Fe-S] proteins as the primary targets of cobalt ions. It was shown that cobalt competes out iron during [Fe-S] cluster synthesis in metabolically essential proteins, and inactivates [Fe-S] enzymes. Cobalt stress resulted in oxidative stress, perturbation of iron homeostasis and decreased sulfur assimilation. Moreover, genes related to the biosynthesis of [Fe-S] gene clusters and cobalt efflux were upregulated, and genes involved in the transport of iron and nickel ions were downregulated in *E.coli* cells, when exposed to cobalt ions. This suggested that the transporters for nickel and iron were possibly used by cobalt ions to enter the cells (Ranquet et al., 2007; Barras and Fontecave, 2011). As reduced iron results in oxygen toxicity by producing hydroxyl radicals in the Fenton reaction, life under aerobic conditions requires a strictly regulated iron metabolism. Consequently, a transcriptomic study with *R. sphaeroides* showed that many genes of iron metabolism are induced upon oxidative stress (Peuser et al., 2012). Thus, the observed cross-resistance against iron stress may also provide survival and growth advantage to our evolved strain G7 under aerobic conditions. It is also important to note that *R. sphaeroides* may have increased its activities related to iron transport and metabolism, as a response and resistance mechanism against cobalt stress, considering the fact that both Co^2+^ and Fe^2+^ are divalent cations and the existing cellular mechanism for Fe^2+^ response can be used by the cells when they are exposed to a less common ion, such as Co^2+^, as reported for *E. coli* previously (Barras and Fontecave, 2011). We have previously observed this behaviour also in a cobalt-resistant, evolved yeast (*S. cerevisiae*) strain that was cross-resistant to iron and nickel stress, and many genes implicated in iron metabolism under the control of the transcriptional activator Aft1p were found to be highly upregulated in that cobalt-resistant *S. cerevisiae* strain (Çakar et al., 2009; Alkım et al., 2013). In addition, a nickel-resistant, evolved *S. cerevisiae* strain also showed cross-resistance against cobalt and iron stress, along with the upregulation of many Aft1p-regulated genes (Küçükgöze et al., 2013), indicating that resistance against cobalt and nickel stress may be associated with the iron response and resistance mechanism in *S. cerevisiae*. The observed cross-resistance of the G7 strain against iron and nickel stress may also be related to a similar, common resistance mechanism in *R. sphaeroides* for cobalt, iron and nickel stresses. The nickel cross-resistance of G7 was also high, as it could survive 2.4 mM NiCl_2_ stress. In a previous study with nickel-resistant bacteria isolated from industrial wastewater, the maximum tolerable nickel concentrations of some isolated species were 1 mM for *Moraxella bovis*, 2 mM for *Acinetobacter lwoffii, Providencia stuartii* and *Branhamella catarrhalis* (Alboghobeish et al., 2014), implying that our G7 strain’s nickel resistance is comparable to that of the bacterial isolates from heavy metal-rich industrial wastewater. Thus, a comparative transcriptomic analysis of our cobalt-resistant evolved strain is likely to provide differentially expressed genes related to Fe-S clusters and iron and nickel transporters, similar to the transcriptomic results with cobalt-exposed *E.coli* (Barras and Fontecave, 2011).

The observed cross-resistance of G7 against Mg^2+^ stress may possibly be associated with the competition of Co^2+^ with Mg^2+^ in ion transport (Losurdo et al., 2010), as high Mg^2+^ concentrations were shown to rescue *R. sphaeroides* from Co^2+^ toxicity. It has been previously shown that Co^2+^ interferes with the extracellular immobilization of Mg^2+^ and its transport across the membrane, implying that Co^2+^ and Mg^2+^ share binding sites on the cell envelope and ion transport systems (Italiano et al., 2009).

The cobalt-resistant, evolved strain G7 was also highly cross-resistant against 0.5 M NaCl stress. A previous study with *R. sphaeroides* strain R26 showed that the strain was halotolerant when grown under anaerobic conditions. It was also suggested that *R. sphaeroides* could be used for the bioremediation of saline and hypersaline polluted environments, as an environmentally friendly and cost-efficient strategy (Labarile et al., 2021). Our results revealed that the evolved G7 strain of *R. sphaeorides* was halotolerant under aerobic growth conditions, supporting its potential in bioremediation of saline and hypersaline environments that are polluted by various metals and/or chemicals.

Our Flame Atomic Absorption Spectrometry results revealed that the cobalt-resistant, evolved strain G7 had a significantly higher cobalt content than RS (Table 2). It has been previously reported that, when grown in the presence of 5 mM Co^2+^, *R. sphaeroides* cells could bind cobalt ions. A previous X-ray absorption spectroscopy study showed that cobalt was bound to the carboxylate groups in the soluble portion of *R. sphaeroides* cells. Carboxylate and sulfonate moieties were shown to be involved in the binding of the bivalent ion in the case of the cell envelope fraction, where the sulfonate functional groups resulted from the sulfolipids of the *R. sphaeroides* cell envelope (Belviso et al., 2013). In the case of our evolved strain G7, it is likely that the same groups may play an active role in binding cobalt ions which remains to be investigated further by X-ray absorption spectroscopy and transmission electron microscopy (TEM) analyses to determine the final cellular destination of cobalt ions. A similar study that involved X-ray absorption spectroscopy and TEM analysis of a cobalt-resistant *Geobacter sulfurreducens* strain showed that cobalt was immobilized on the cell surface. Transcriptomic analysis of that *G. sulfurreducens* strain also revealed various changes such as upregulation of several metal efflux pumps, downregulation of non-essential proteins with metals and thiol groups that Co^2+^ preferentially targets, cell envelope modification to increase metal resistance and biofilm formation, and activation of sensory and regulatory proteins involved in detoxification (Dulay et al., 2020). Thus, it is likely that Co^2+^ may also be immobilized on the cell surface of our evolved strain, associated with the cell envelope.

The ability of the G7 strain to hold high amounts of cobalt ions, and its high resistance against cobalt and many other stressors indicate its high bioremediation potential.

Comparative whole genome re-sequencing results of the cobalt-resistant, evolved strain G7 revealed two missense mutations in the mviN gene that encodes a putative virulence factor. In *E. coli*, the mviN gene codes for an essential protein for peptidoglycan synthesis, MviN (MurJ), a kind of integral membrane protein, and it has a role as a flippase which transports lipids from the exoplasmic surface toward the cytosolic surface (Ruiz, 2008). As a kind of lipid II flippase, MviN translocates peptidoglycan precursors (lipid II) across the cytoplasmic membrane to the cytoplasm (Ruiz, 2016). Similarly, MviN was also expected to function as a flippase in *Mycobacterium tuberculosis*, as the deficiency of it resulted in the accumulation of peptidoglycan precursors in the cytoplasm (Arora et al., 2018). The two missense mutations in the mviN gene of our evolved strain might have resulted in changes in the cell membrane and/or peptidoglycan that possibly contributed to an increase in cobalt resistance and robustness of G7.

The missense mutation observed in RSP_2320 may also be important for the cobalt resistance of our evolved strain. RSP_2320 encodes a Tripartite ATP-independent periplasmic (TRAP) family transporter, periplasmic binding protein (Callister et al., 2006). TRAP transporters use ionoelectrochemical gradients to transport molecules actively across a membrane in the same direction (Rosa et al., 2018). In a previous study, comparative transcriptomic and genomic analyses of cobalt-sensitive mutant strains of *R. sphaeorides* obtained by transposon mutagenesis were performed. It was found that RSP_0097 encoding ‘TRAP-type C4-dicarboxylate transport system periplasmic component’ and RSP_1419 encoding ‘TRAP-T family transporter small inner membrane subunit’ were downregulated in a cobalt-sensitive mutant strain (Volpicella et al., 2014). Comparative photosynthetic membrane proteome analysis of *R. sphaeroides* under 5 mM cobalt stress and control conditions also identified the TRAP di-carboxylate family transporter DctP subunit as an upregulated protein (Italiano et al., 2011). More recently, the lack of the TakP transporter, a member of the TRAP family of transporters, was shown to lead to selenite-induced oxidative stress in *R. sphaeroides* (Adnan et al., 2021). Based on these findings, it can be suggested that the identified missense mutation in RSP_2320 associated with a TRAP family transporter might have increased the TRAP transporter activity of our evolved strain G7, which may have contributed to its increased resistance to cobalt and other stressors. The exact mechanism, however, is yet to be investigated.

Another missense mutation was observed in the rpoD gene encoding the RNA polymerase, sigma 70 subunit RpoD. In a previous study with cobalt-sensitive *R. sphaeroides* mutants obtained by transposon mutagenesis, it was found that the rpoH2 gene (RSP_0601) encoding a DNA-directed RNA polymerase sigma subunit was upregulated in a cobalt-sensitive mutant strain (Volpicella et al., 2014). The exact role of RNA polymerase in the cobalt resistance of *R. sphaeroides* is yet to be investigated in more detail.

The nifB gene encoding the FeMo cofactor biosynthesis protein of NifB family in *R. sphaeroides* also had a missense mutation in our evolved strain G7. Bacterial nif genes are nitrogen fixation regulatory genes and have roles in encoding the nitrogenase enzyme complex components (Dahal et al., 2021). Volpicella et al. (2014) showed that genes that code for the nitrogenase subunit NifH (ATPase), bchX (RSP_0262) and bchL (RSP_0288), and genes encoding a nitrogenase molybdenum-iron protein alpha and beta chain, bchZ (RSP_0260) and bchY (RSP_0261), were upregulated in a cobalt-sensitive *R. sphaeroides* strain obtained by transposon mutagenesis. These findings indicate that the nitrogenase enzyme complex may have a potential role in the cobalt resistance of *R. sphaeroides*.

The nhaD gene that encodes a sodium/proton antiporter protein of the NhaD family in *R. sphaeroides* also had a missense mutation in our cobalt-resistant, evolved strain G7. In a previous study, RSP_2638 that encodes a Ca^2+^/Na^2+^ antiporter was found to be upregulated in a cobalt-sensitive *R. sphaeroides* strain (Volpicella et al., 2014). It is known that sodium/proton-antiporters (Nha) are important in pH and Na^+^-homeostasis (Kurz et al., 2006). Thus, the observed high NaCl cross-resistance of G7 may be related to a possibly activated NhaD antiporter and the changes in transporter activities in G7 may also be related to its resistance to cobalt and many other stressors.

Another missense mutation was observed in RSP_3988 that codes for a putative glycosyltransferase. In a previous study, proteomic characterization of the *R. sphaeroides* photosynthetic membrane was performed, and 28 proteins were identified as intracytoplasmic membrane (ICM)-associated proteins, most likely localized in the periplasm, cytoplasm or outer membrane of *R. sphaeroides* cells. ICM is an inducible membrane that has an important role in bacterial photosynthesis, harvesting light energy, separation of primary charges and electron transport. Interestingly, the product of RSP_3988 was also present among these 28 ICM-associated proteins, along with six other proteins that are periplasmic subunits of ABC or TRAP transporters (Zeng et al., 2007). The exact role of RSP_3988 in *R. sphaeroides* energy metabolism and cobalt resistance needs to be investigated in more detail.

ABC transporters are conserved from bacteria to humans, and are powered by ATP to move substrates across cellular membranes. They are strongly regulated to balance essential nutrient needs and substrate toxicity effects, and they consist of both exporters and importers. ABC transporters are necessary for the transport of various compounds such as antibiotics, lipids, and proteins. Moreover, various ABC transporters have been reported to transport diverse metals such as zinc, manganese, iron, nickel and cobalt (Tanaka et al., 2018). An ABC transporter (cntABCDF) responsible for cobalt and nickel transport was identified in the major opportunistic pathogen *Staphylococcus aureus*. It also affected urease activity and bacterial colonization in systemic and urinary tract infection models, indicating the role of this cobalt and nickel transporter in the virulence of *S. aureus* (Remy et al., 2013). Similarly, another ABC transporter (FecDE and CeuE) that contributes to nickel and cobalt acquisition was identified in the bacterial pathogen *Helicobacter mustelae,* where the *fecD* mutant had decreased cobalt levels and improved cobalt resistance (Stoof et al., 2010). As reported previously, in a cobalt-sensitive *R. sphaeroides* strain mutated in the RSP_7363 gene that encodes a hypothetical protein, an ABC sugar transporter was significantly downregulated. It was suggested that the ABC sugar transporter may provide an additional energy source that may help the mutant strain to overcome cobalt stress, as the mutant strain was not inhibited when grown under photosynthetic conditions that can provide an additional energy source instead of the impaired ABC sugar transporter (Volpicella et al., 2014). A different ABC sugar transporter of *R. sphaeroides* was found to play a role in its adaptation to high concentrations of selenite ions: a *R. sphaeroides* mutant strain deficient in *smoK* gene encoding an ABC transporter for the uptake of sugar alcohols could tolerate selenite. It was suggested that selenite uptake into *R. sphaeroides* cells may occur by a sugar alcohol transporter in *R. sphaeroides* (Bebien et al., 2001). Similarly, in the soil bacterium *Sinorhizobium meliloti* that can form N_2_-fixing root nodules on its plant host alfalfa, an ABC sugar transporter was shown to play a role in the regulation of potassium transport and response to alkali stress conditions (Lin et al., 2009). It was also reported that the gene cluster *cbtJKL* encodes an ABC-type cobalt-transport system in *S. meliloti*, which was required for the growth of free-living cells at required trace element cobalt concentrations, but not required for symbiotic N_2_ fixation (Cheng et al., 2011). Thus, in our cobalt-resistant *R. sphaeroides* strain, it is also likely that the observed cobalt resistance may be associated with differential expression of some ABC transporters as reported previously (Volpicella et al., 2014), which remains to be investigated by comparative transcriptomic analyses.

In our evolved strain G7, another missense mutation was detected in RSP_4277 that encodes a transcriptional regulator of MerR family. Zeller et al. (2007) investigated the regulatory functions of the OxyR protein in *R. sphaeroides*. OxyR is known as a regulatory protein of H_2_O_2_ response in bacteria. Comparative transcriptomic analysis results of wild-type and *OxyR* mutant *R. sphaeroides* strains in the presence and absence of 1 mM H_2_O_2_ stress (with 7 min exposure time) revealed that multiple genes were upregulated both in the wild-type and OxyR mutant strains, including RSP_4277. It was concluded that additional regulatory pathways other than OxyR may play a role in the oxidative stress response of *R sphaeroides*. Thus, the RSP_4277 missense mutation observed in our evolved strain may also be related to its oxidative stress response and/or cobalt resistance.

The mbfA gene (RSP_0850) codes for a membrane-bound ferritin in *R. sphaeroides*, and is associated with iron storage (Peuser et al., 2012). Thus, the missense mutation in the mbfA gene of our evolved strain G7 may also contribute to its resistance against cobalt and iron stresses.

In this study, we have identified novel mutations that may play an important role in the cobalt-resistance and robustness of *R. sphaeroides*. In a previous study with two cobalt-sensitive *R. sphaeroides* strains obtained by transposon mutagenesis, the insertion sites were identified in other genes such as RSP_7363, encoding a hypothetical protein, which are not mutated in our cobalt-resistant, evolved strain (Volpicella et al., 2014). These findings imply the multigenic and complex molecular basis of cobalt-resistance in *R. sphaeroides*. Volpicella et al. (2014) also reported that all insertions were detected outside the chromosome II, supporting a previous statement that the genes with essential roles are located in the chromosome II of *R. sphaeroides* (Choudhary et al., 1994). In our cobalt-resistant evolved strain, however, we have detected three missense mutations (two in RSP_3791 and one in RSP_3478) and multiple synonymous mutations (in RSP_3791) in chromosome II, in addition to some other mutations in chromosome I, Plasmid B (in RSP_3988) and Plasmid E (RSP_4277) (Table 3).

It is important to note that, in our cobalt-resistant evolved strain, two missense and eight synonymous mutations were identified in RSP_3791, encoding a hypothetical protein. In addition, many genes encoding hypothetical proteins were differentially regulated in a cobalt-sensitive mutant *R. sphaeroides* strain obtained by transposon mutagenesis (Volpicella et al., 2014). Thus, the hypothetical protein encoded by RSP_3791 and other hypothetical proteins may play a crucial role in the cobalt resistance of *R. sphaeroides* which is yet to be investigated.

The purpose of performing comparative whole-genome re-sequencing of the cobalt-resistant, evolved *R. sphaeroides* strain was to identify genomic variations in the evolved strain that may be potential molecular targets for cobalt resistance. Our results showed that the genomic variations identified in the evolved strain do not seem to belong to the genes that are directly related to cobalt accumulation (Table 3). A previous study on cobalt-sensitive mutants of *R. sphaeroides* obtained by transposon mutagenesis also revealed mutations in genes that seem not to be directly related to cobalt accumulation or in genes encoding hypothetical proteins. It was, however, found that, at transcriptomic level these mutations can reduce the expression of ABC sugar transporter operon subunits, indicating that cobalt response is associated with energy metabolism (Volpicella et al., 2014). It is important to note that cobalt resistance and stress resistance, in general, are multigenic and complex traits, which usually involve multiple, seemingly unrelated molecular factors and/or pathways, as we have also observed in our previous studies with yeast (Alkım et al., 2013; Balaban et al., 2020; Terzioğlu et al., 2020). Considering that the *R. sphaeroides* strain was not exposed to random/chemical mutagenesis prior to evolutionary engineering, it is likely that many of the observed mutations in our evolved strain that occurred only under the selective pressure of cobalt stress may be related to cobalt resistance, or they may influence the expression levels of cobalt-associated genes, at the transcriptomic level.

## 5 Conclusion

In this study, we have obtained a highly cobalt-resistant and genetically stable *R. sphaeroides* strain by using evolutionary engineering. The evolved strain could resist as high as 15 mM CoCl_2_ stress, which, to the best of our knowledge, has not been reported so far. In addition, the evolved strain could accumulate significantly higher amounts of cobalt than the reference strain. It was also cross-resistant to many other stressors, which indicate a high potential for its use as a robust and multi-stress resistant *R. sphaeroides* strain in bioremediation applications. Comparative genomic analyses of the evolved strain identified several unique mutations in multiple genes with diverse functions and particularly in genes encoding hypothetical proteins, such as RSP_3791, the exact role of which remains to be investigated in detail to fully understand the complex, multigenic basis of cobalt resistance in *R. sphaeroides*. To this end, comparative transcriptomic and/or proteomic analyses of the evolved strain, and reverse engineering studies to introduce the observed genetic variations into the reference strain by genome editing and testing the resulting strains for cobalt resistance are planned as future studies.

## Data availability statement

The datasets presented in this study can be found in online repositories. The names of the repository/repositories and accession number(s) can be found at: https://www.ncbi.nlm.nih.gov/, BioProject PRJNA1047122.

## Author contributions

GA: Project administration, Writing – review & editing, Data curation, Methodology, Writing – original draft. CH: Data curation, Methodology, Project administration, Writing – original draft, Writing – review & editing. HC: Data curation, Methodology, Writing-original draft. MA: Data curation, Methodology, Writing – original draft. AT: Data curation, Methodology, Writing – original draft, Project administration, Writing – review & editing. MT: Project administration, Writing – review & editing, Conceptualization, Funding acquisition, Supervision. ZÇ: Conceptualization, Funding acquisition, Project administration, Supervision, Writing – review & editing.
