# Supplementary figures and images for: Evolutionary engineering and molecular characterization of cobalt-resistant Rhodobacter sphaeroides
Source: Front Microbiol. 2024 Jun 27;15:1412294. doi: 10.3389/fmicb.2024.1412294 (PMC11236759; doi:10.3389/fmicb.2024.1412294)

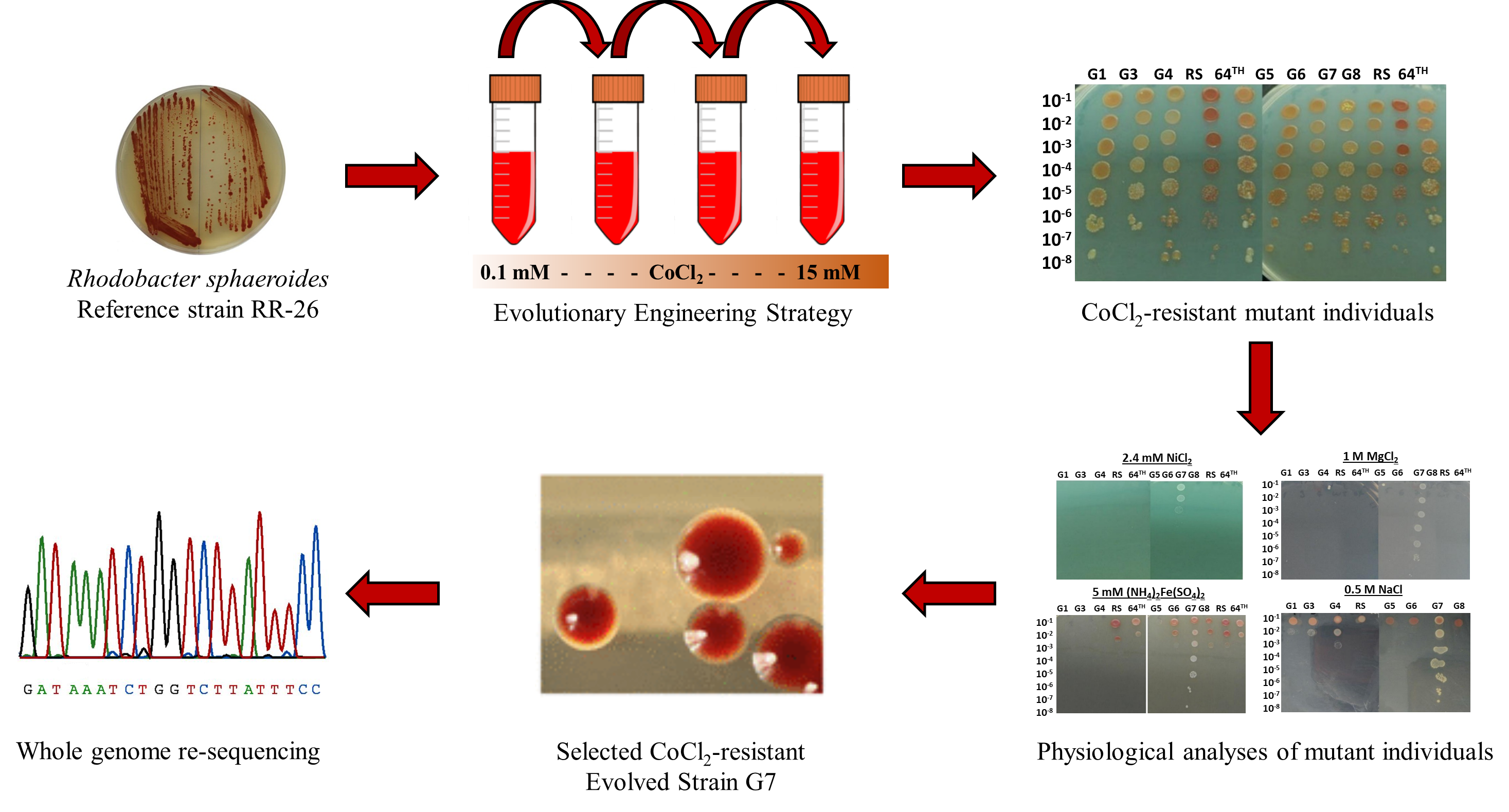

Supplement: Supplementary file 1 [file Graphical_Abstract.TIF]

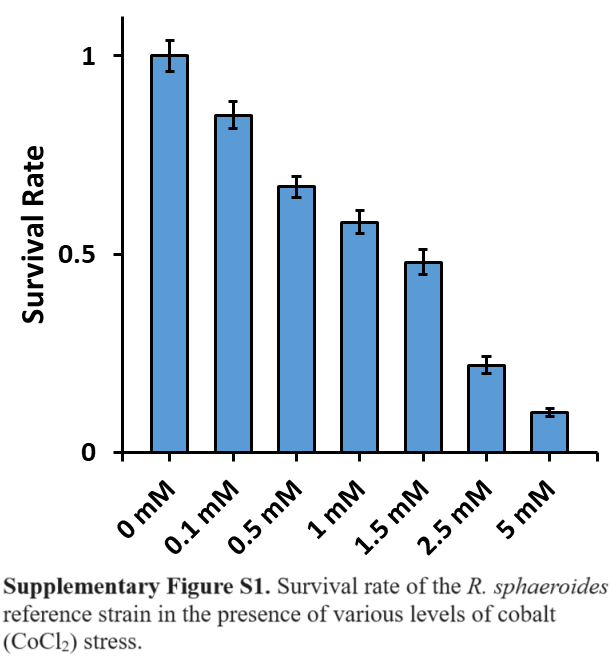

Supplement: Supplementary file 2 [file Supplementary_Figure_S1.TIF]
